# Supplementary material for: CuMoxW(1-x)O4 Solid Solution Display Visible Light Photoreduction of CO2 to CH3OH Coupling with Oxidation of Amine to Imine
Source: Nanomaterials (Basel). 2020 Jul 3;10(7):1303. doi: 10.3390/nano10071303 (PMC7408418; doi:10.3390/nano10071303)
Supplement: Supplementary file 1 [file nanomaterials-10-01303-s001.pdf]

$CuMo_xW_{(1-x)}O_4$ Solid Solution Display Visible Light Photoreduction of CO_2 to CH_3OH Coupling With Oxidation of Amine to Imine

Chao Luo [1], Tian Yang [3], Qianfei Huang [2], Xian Liu [3], Huan Ling [1], Yuxin Zhu [1], Guoming Xia [1], Wennan Zou [1,*] and Hongming Wang [1,*]

[1] Institute for Advanced Study, Nanchang University, Nanchang 330031, China; luochao@ncu.edu.cn (C.L.); huanling_ncu@126.com (H.L.); ncu_zhuyuxin@163.com (Y.Z.); gmxia_ncu@126.com (G.X.)

[2] School of Information Engineering, Jiangxi Modern Polytechnic College, Jiangxi 330095, China; huangqianfei1015@hotmail.com

[3] College of Chemistry, Nanchang University, Nanchang 330031, Jiangxi, China; yangtian_92@163.com (T.Y.); lzhnxydlx@163.com (X.L.)

* Correspondence: zouwn@ncu.edu.cn (W.Z.); hongmingwang@ncu.edu.cn (H.W.)

The standard curve method was applied to carry out the quantitative analysis using benzylamine, methanol and N-benzylidenebenzylamine as standard sample, respectively.

The conversion rate of benzylamine were obtained according to the internal standard curve:

$$m_{t0} = 1.9451 \times 10^{-4} \times A_{t0} - 0.027, \quad (1)$$

$$Con.\% = (m_0 - m_{t0})/m_0 \times 100\%, \quad (2)$$

m_0 = 0.9813mg, slope:1.9451×10^{-4}, intercept: -0.027, m_0: the mass of benzylamine, A_{t0}: Gas chromatographic peak area of benzylamine at time t, m_{t0}: the mass of benzylamine at time t.

The yield of CH_3OH were obtained according to the formula:

$$Y_t = 0.34482 \times A_{t1} + 0.012 \quad (3)$$

A_{t1}: Gas chromatographic peak area of CH_3OH at time t, Y_t: The yield of CH_3OH at time t, units: μmol, slope: 0.34482, intercept 0.012.

The selectivity of N-benzylidenebenzylamine were obtained according to the formula:

$$m_{t1} = 2.4091 \times 10^{-4} \times A_{t1} - 0.016, \quad (4)$$

$$Sel.\% = m_{t1}/(m_0 - m_{t0}), \quad (5)$$

slope: 2.4091 × 10^{-4}, intercept: −0.016, m_{t1}: the mass of N-Benzylidenebenzylamine at time t, A_{t1}: Gas chromatographic peak area of N-benzylidenebenzylamine at time t.

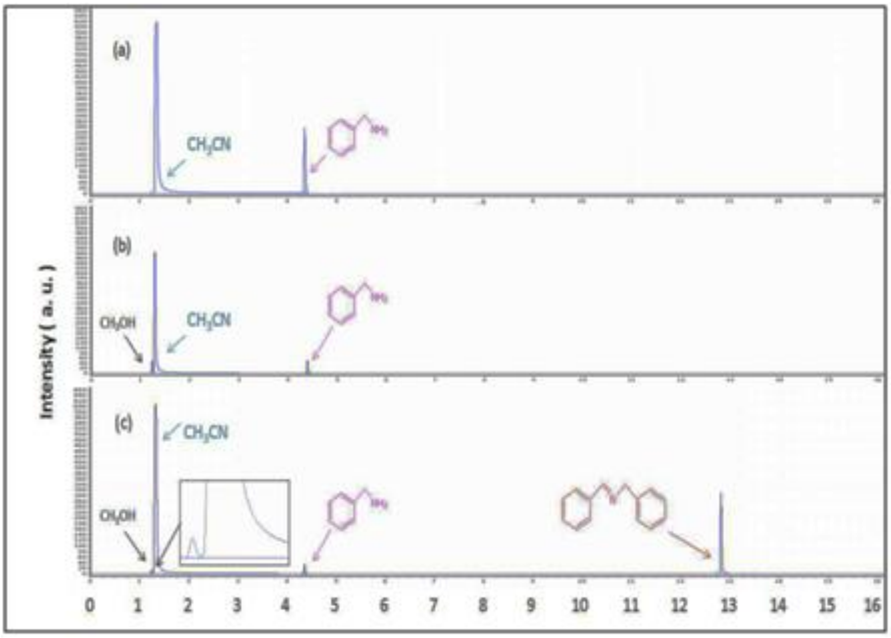

Figure S1. GC chromatograms of (**a**) before irradiation and (**b**) the stand CH_3OH and benzylamine in CH_3CN and (**c**) product (CH_3OH) after irradiation for 10 hours with the partial enlarged drawing.

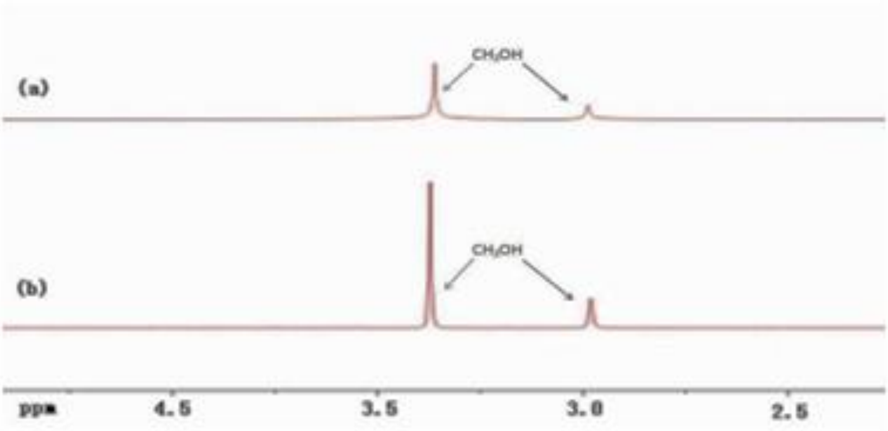

Figure S2. The ^{1}H NMR spectrums of (**a**) the product (CH_3OH) photocatalyzed by $CuW_{0.7}Mo_{0.3}O_4$ (x = 0.7) after reaction for 10 hours and (**b**) the stand CH_3OH.

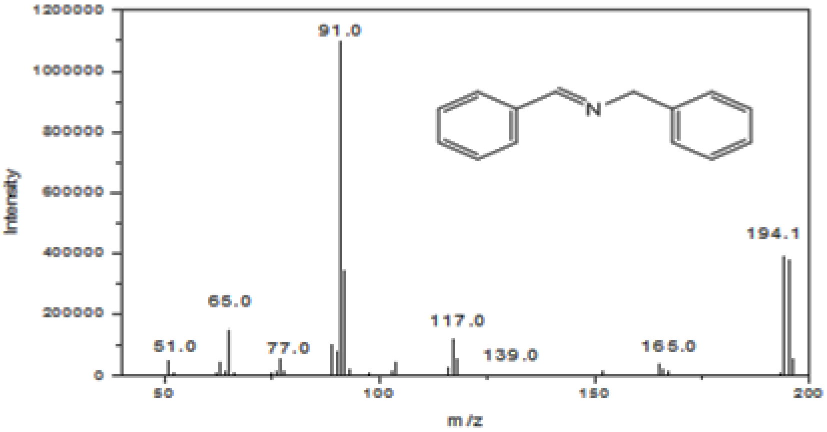

Figure S3. the MS of Products photocatalyzed by $CuW_{0.7}Mo_{0.3}O_4$ (x = 0.7) after irradiation for 10 hours.

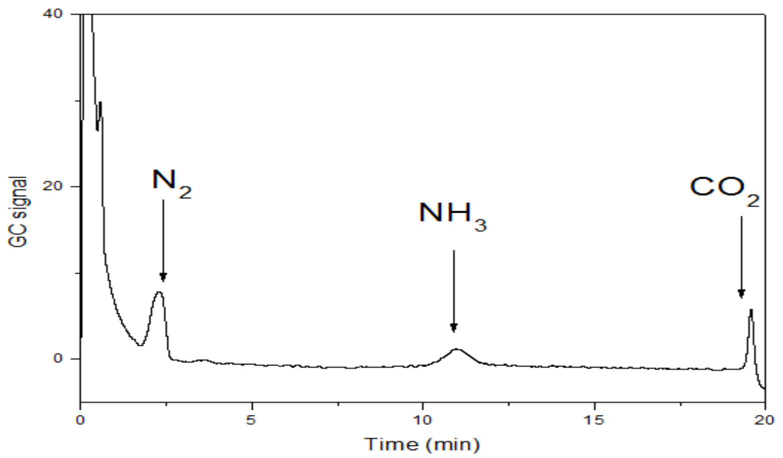

Figure S4. GC chromatograms of gas phase products after irradiation for 10 hours.

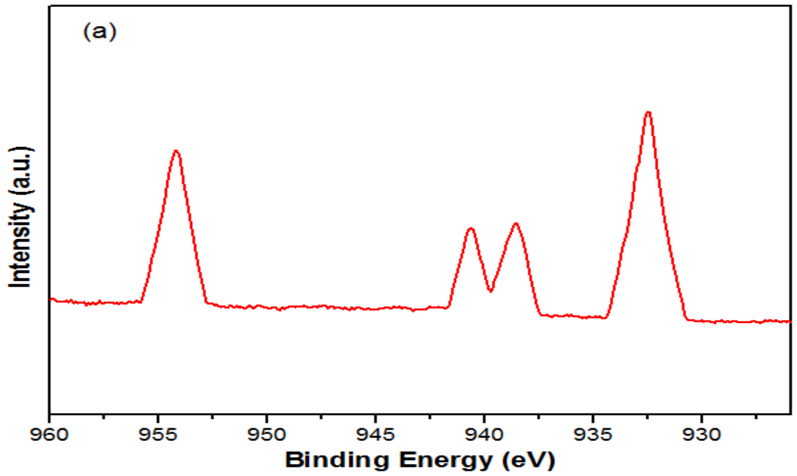

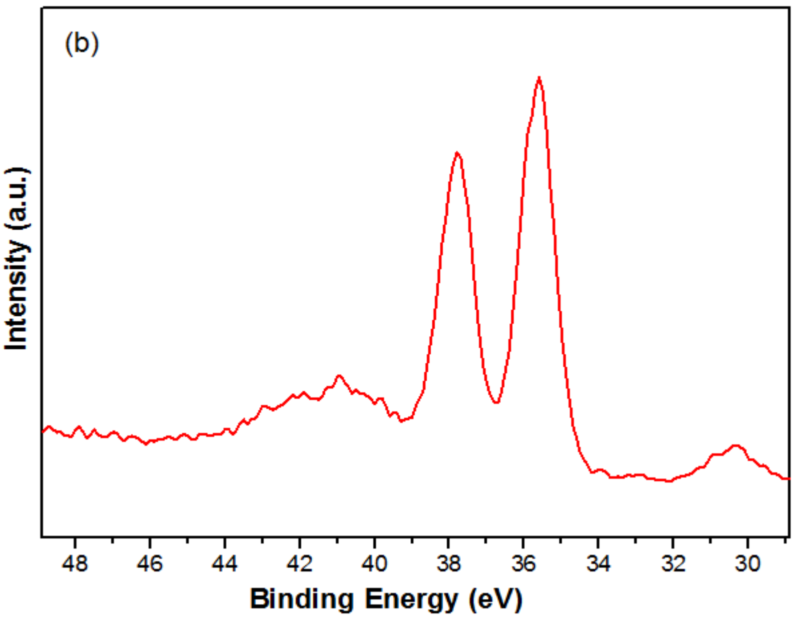

Figure S5. XPS spectra of (**a**) Cu_{2p} and (**b**) W_{4f} of photocatalysts.
